# Supplementary figures and images for: Impact of ultraviolet germicidal irradiation on new silicone half-piece elastometric respirator (VJR-NMU) performance, structural integrity and sterility during the COVID-19 pandemic
Source: PLoS One. 2021 Oct 14;16(10):e0258245. doi: 10.1371/journal.pone.0258245 (PMC8516203; doi:10.1371/journal.pone.0258245)

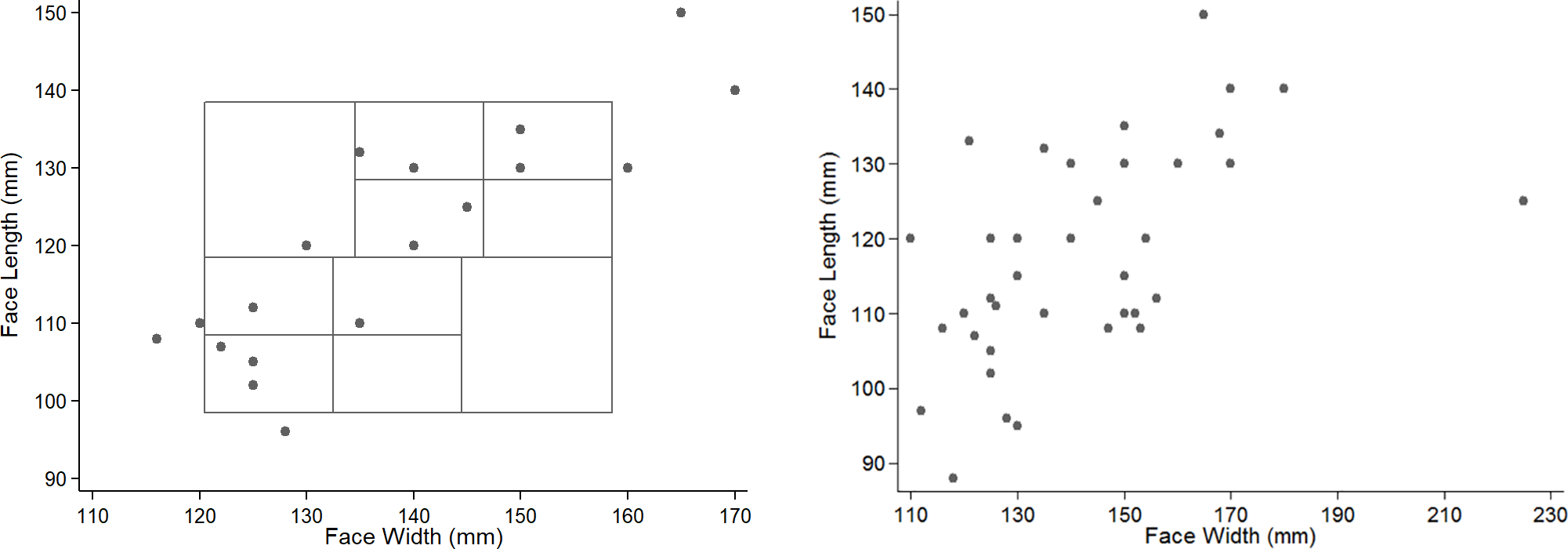

Supplement: S1 Fig — a Bivariate model of face dimension distribution. b Scatter bivariate distribution of face dimension. (TIF) [file pone.0258245.s002.tif]

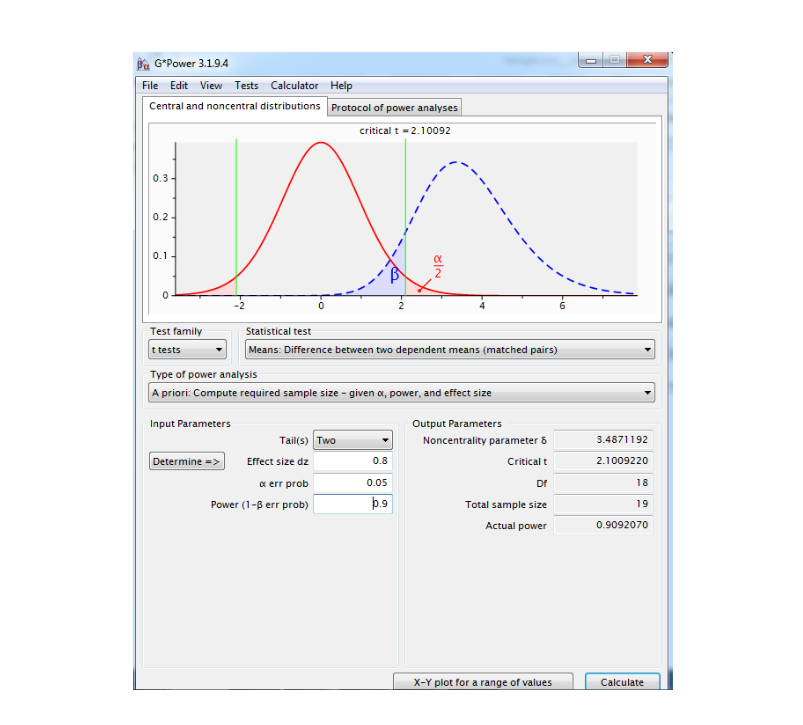

Supplement: S2 Fig — (TIF) [file pone.0258245.s003.tif]

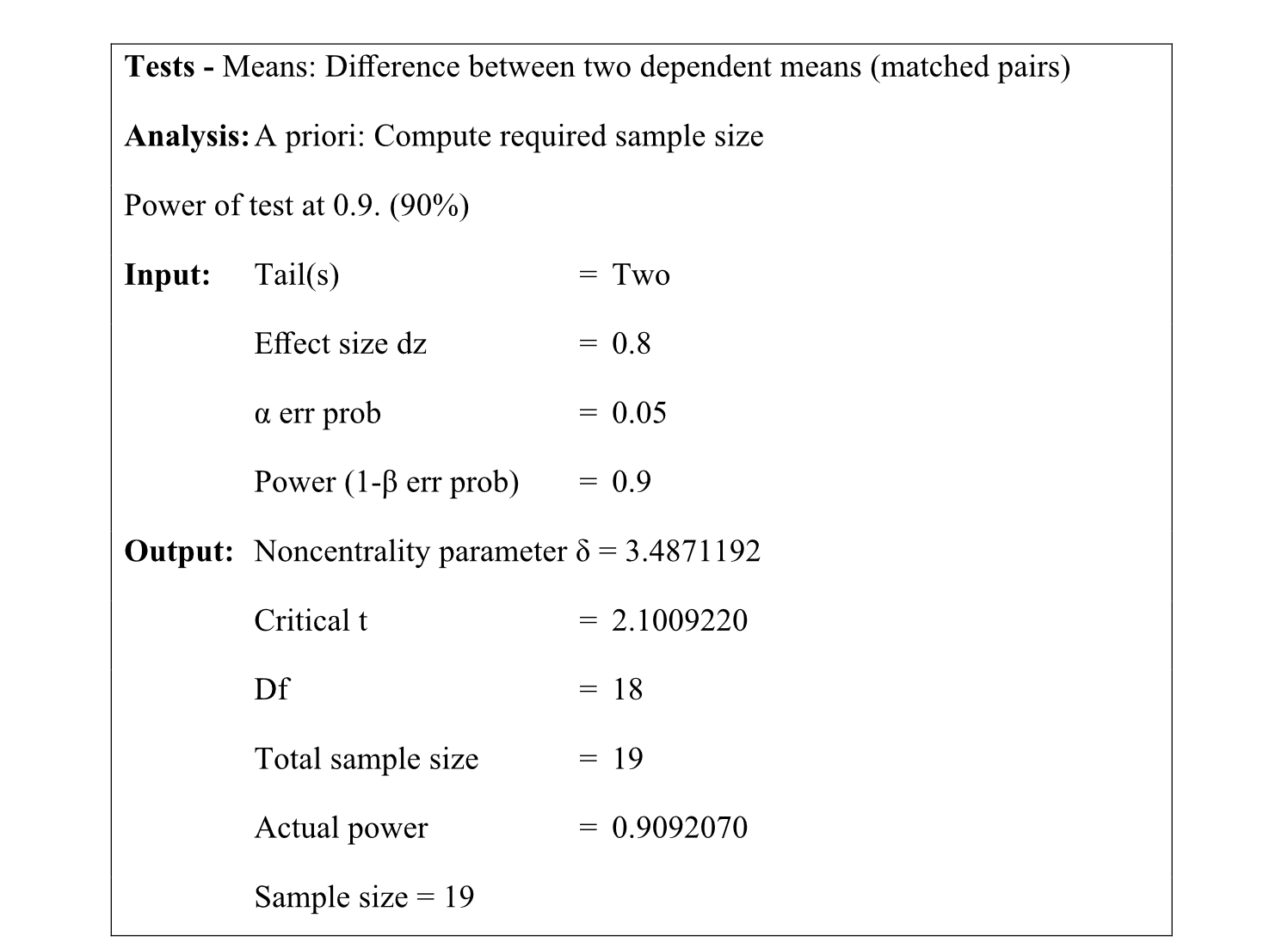

Supplement: S3 Fig — (TIF) [file pone.0258245.s004.tif]

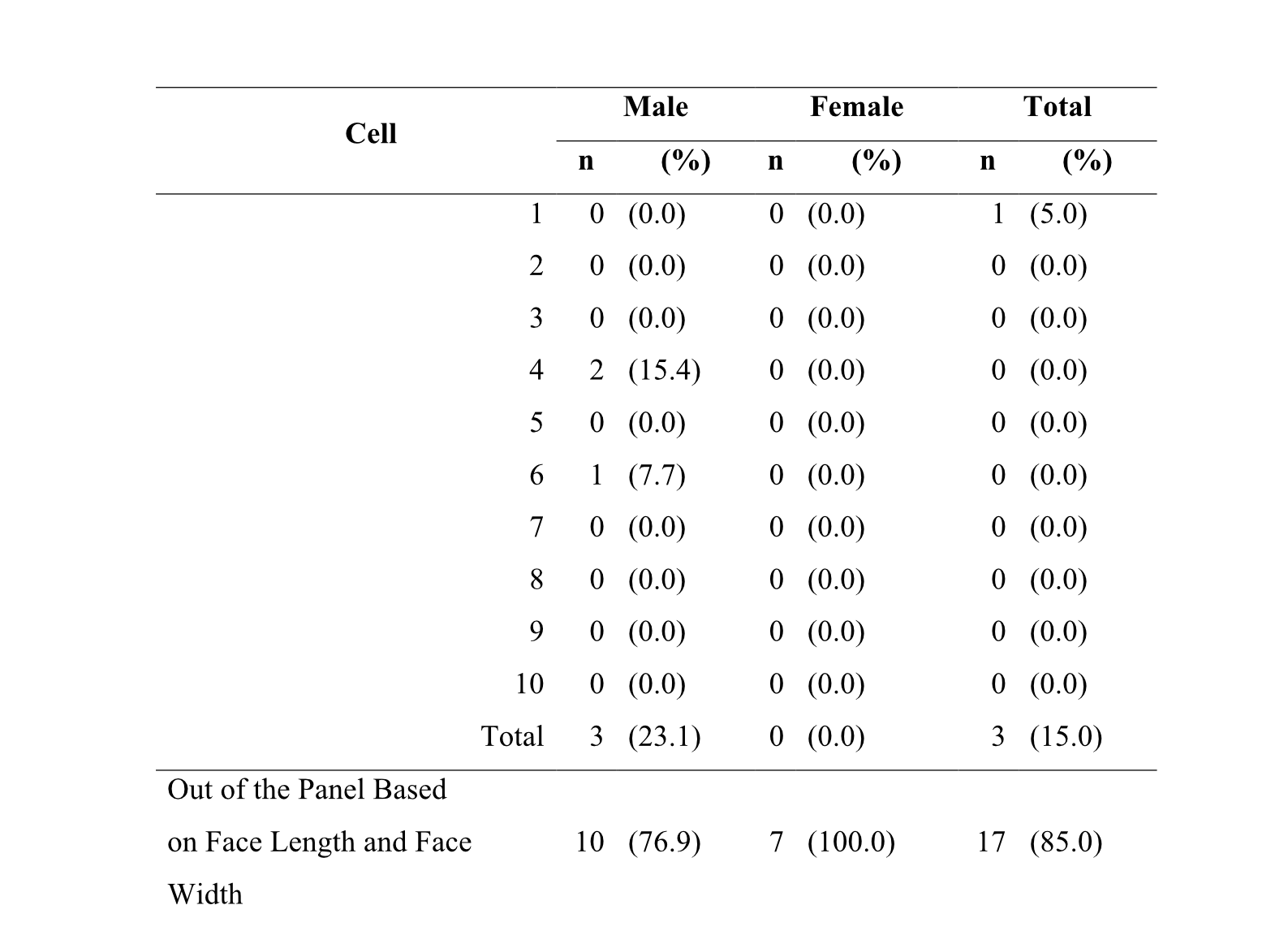

Supplement: S1 Table — (TIF) [file pone.0258245.s005.tif]
