# Supplementary material for: Impact of ultraviolet germicidal irradiation on new silicone half-piece elastometric respirator (VJR-NMU) performance, structural integrity and sterility during the COVID-19 pandemic
Source: PLoS One. 2021 Oct 14;16(10):e0258245. doi: 10.1371/journal.pone.0258245 (PMC8516203; doi:10.1371/journal.pone.0258245)
Supplement: S1 File — (DOCX) [file pone.0258245.s006.docx]

The experiment by BIOTEC (National Center for Genetic and Biotechnology) team

By Dr. Anan Jongkaewattana

The BIOTEC team cultured virus on N95 mask by dropping porcine epidemic diarrhea virus ( PEDV) which is non Pathogenic in Human 200 microliter onto the middle of N95 two hours before UVC irradiation.The experiment was carried out at different doses and time at 70 microWatt-sec/cm^2 .^ or more.They found that the virus could not survive at this UVC dosage .The unexposed control still find the virus particle ( Pink color **)**

|  | 1 min | 5 min | 10 min | 20 min |
| --- | --- | --- | --- | --- |
| Upper | 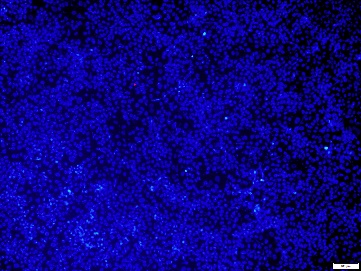 | 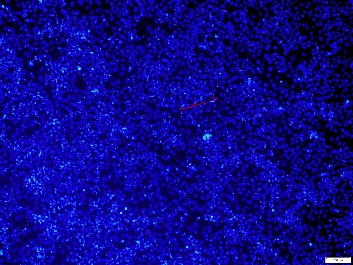 | 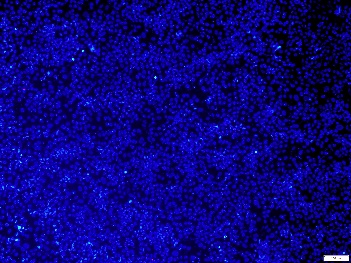 | 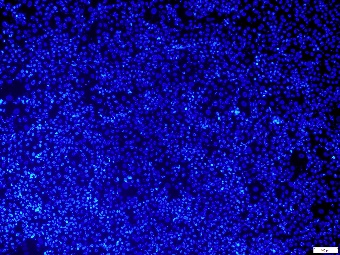 |
| Lower | 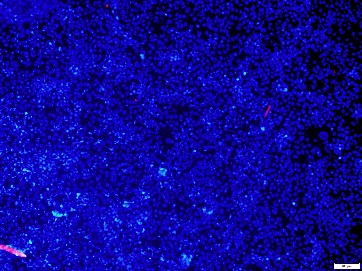 | 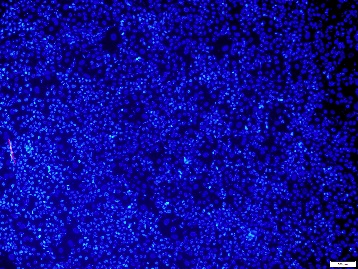 | 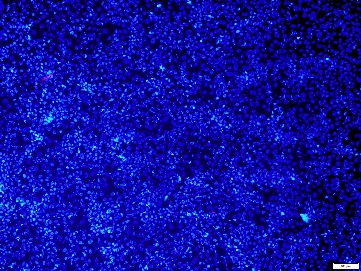 | 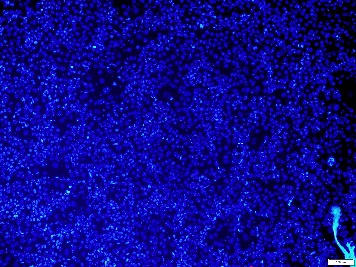 |
|  | 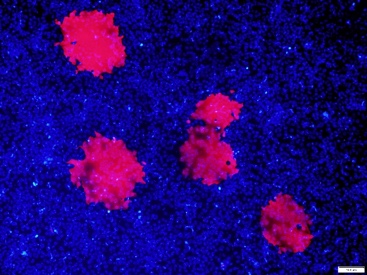 | Unexposed control |  |  |

**Tensile System Specification**

Mechanical Tester (Universal Testing (static Type, 100N, 10 kN) INSTRON4502

- **Test Method :** Tension test
- **Instrument :** Instron 55R4502
- **Load Cell :** 100 N
- **NCTC :** 62-N03-3646
- **Test Conditions. Speed :** 500 mm./min.
- **Grip Distance :** 40 mm.
- **Gauge Length :** None
- **Test Temperature :** 23.0 Degree Celsius
- **Test Humidity :** 50 % R.H.
- **Operated by :** *Miss Natsuda Palawat*; NCTC, NSTDA
