# Supplementary material for: Impact of ultraviolet germicidal irradiation on new silicone half-piece elastometric respirator (VJR-NMU) performance, structural integrity and sterility during the COVID-19 pandemic
Source: PLoS One. 2021 Oct 14;16(10):e0258245. doi: 10.1371/journal.pone.0258245 (PMC8516203; doi:10.1371/journal.pone.0258245)
Supplement: S1 Appendix — (DOCX) [file pone.0258245.s007.docx]

**Appendix**

| **Table 1** Comparison between before and after decontamination | | | |  | |  | |  | |
| --- | --- | --- | --- | --- | --- | --- | --- | --- | --- |
| **Testing** | **Before** | | **After** | | | | **p-value*** | |  |
|  | **Median** | **(IQR)** | **Median** | | **(IQR)** | |  |  |  |
| Decontamination with 70% Alcohol (10 min) |  |  |  | |  | |  | |  |
| Bacteria outside the mask (CFU/m^3^) | 1 | (0-3) | 0 | | (0-1) | | 0.116 | |  |
| Fungus outside the mask (CFU/m^3^) | 0 | (0-0) | 0 | | (0-0) | | 0.317 | |  |
| Bacteria inside the mask (CFU/m^3^) | 0 | (0-1.5) | 0 | | (0-0) | | 0.009 | |  |
| Fungus inside the mask (CFU/m^3^) | 0 | (0-0) | 0 | | (0-0) | | 0.317 | |  |
| Bacteria at filter area (CFU/m^3^) | 0.5 | (0-3) | 0 | | (0-0) | | 0.002 | |  |
| Fungus at filter area (CFU/m^3^) | 0 | (0-0) | 0 | | (0-0) | | NA | |  |
| Decontamination with UVC (1 min) |  |  |  | |  | |  | |  |
| Bacteria outside the mask (CFU/m^3^) | 3 | (1.5-5.5) | 0 | | (0-2.5) | | 0.056 | |  |
| Fungus outside the mask (CFU/m^3^) | 0 | (0-0) | 0 | | (0-0) | | NA | |  |
| Bacteria inside the mask (CFU/m^3^) | 0.5 | (0-4.5) | 1 | | (0-4) | | 0.970 | |  |
| Fungus inside the mask (CFU/m^3^) | 0 | (0-0) | 0 | | (0-0) | | NA | |  |
| Bacteria at filter area (CFU/m^3^) | 0 | (0-0.5) | 2 | | (0-3.5) | | 0.009 | |  |
| Fungus at filter area (CFU/m^3^) | 0 | (0-0) | 0 | | (0-0) | | 1.000 | |  |
| Decontamination with UVC (10 min) |  |  |  | |  | |  | |  |
| Bacteria outside the mask (CFU/m^3^) | 3 | (1.5-5.5) | 1 | | (0-4) | | 0.200 | |  |
| Fungus outside the mask (CFU/m^3^) | 0 | (0-0) | 0 | | (0-0) | | 0.317 | |  |
| Bacteria inside the mask (CFU/m^3^) | 0.5 | (0-4.5) | 0 | | (0-1) | | 0.046 | |  |
| Fungus inside the mask (CFU/m^3^) | 0 | (0-0) | 0 | | (0-0) | | NA | |  |
| Bacteria at filter area (CFU/m^3^) | 0 | (0-0.5) | 1 | | (0-2.5) | | 0.055 | |  |
| Fungus at filter area (CFU/m^3^) | 0 | (0-0) | 0 | | (0-0) | | NA | |  |
| Decontamination with UVC (20 min) |  |  |  | |  | |  | |  |
| Bacteria outside the mask (CFU/m^3^) | 3 | (1.5-5.5) | 0 | | (0-1) | | 0.010 | |  |
| Fungus outside the mask (CFU/m^3^) | 0 | (0-0) | 0 | | (0-0) | | 0.317 | |  |
| Bacteria inside the mask (CFU/m^3^) | 0.5 | (0-4.5) | 0 | | (0-0) | | 0.009 | |  |
| Fungus inside the mask (CFU/m^3^) | 0 | (0-0) | 0 | | (0-0) | | 0.157 | |  |
| Bacteria at filter area (CFU/m^3^) | 0 | (0-0.5) | 0 | | (0-1.5) | | 0.071 | |  |
| Fungus at filter area (CFU/m^3^) | 0 | (0-0) | 0 | | (0-0) | | 0.317 | |  |

**Table 2** Comparison between type of decontamination

| **Testing** | **Alcohol 70%** | | **UVC 1 min** | | **UVC 10 min** | | **UVC 20 min** | | **p-value*** |
| --- | --- | --- | --- | --- | --- | --- | --- | --- | --- |
|  | **Median** | **(IQR)** | **Median** | **(IQR)** | **Median** | **(IQR)** | **Median** | **(IQR)** |  |
| **Before decontamination** |  |  |  |  |  |  |  |  |  |
| Bacteria outside the mask (CFU/m^3^) | 1 | (0-3) | 3 | (1.5-5.5) | 3 | (1.5-5.5) | 3 | (1.5-5.5) | 0.113 |
| Fungus outside the mask (CFU/m^3^) | 0 | (0-0) | 0 | (0-0) | 0 | (0-0) | 0 | (0-0) | 0.392 |
| Bacteria inside the mask (CFU/m^3^) | 0 | (0-1.5) | 0.5 | (0-4.5) | 0.5 | (0-4.5) | 0.5 | (0-4.5) | 0.950 |
| Fungus inside the mask (CFU/m^3^) | 0 | (0-0) | 0 | (0-0) | 0 | (0-0) | 0 | (0-0) | 0.392 |
| Bacteria at filter area (CFU/m^3^) | 0.5 | (0-3) | 0 | (0-0.5) | 0 | (0-0.5) | 0 | (0-0.5) | 0.086 |
| Fungus at filter area (CFU/m^3^) | 0 | (0-0) | 0 | (0-0) | 0 | (0-0) | 0 | (0-0) | 0.392 |
| **After decontamination** |  |  |  |  |  |  |  |  |  |
| Bacteria outside the mask (CFU/m^3^) | 0 | (0-1) | 0 | (0-2.5) | 1 | (0-4) | 0 | (0-1) | 0.245 |
| Fungus outside the mask (CFU/m^3^) | 0 | (0-0) | 0 | (0-0) | 0 | (0-0) | 0 | (0-0) | 0.567 |
| Bacteria inside the mask (CFU/m^3^) | 0 | (0-0) | 1 | (0-4) | 0 | (0-1) | 0 | (0-0) | 0.007 |
| Fungus inside the masl (CFU/m^3^) | 0 | (0-0) | 0 | (0-0) | 0 | (0-0) | 0 | (0-0) | 0.108 |
| Bacteria at filter area (CFU/m^3^) | 0 | (0-0) | 2 | (0-3.5) | 1 | (0-2.5) | 0 | (0-1.5) | 0.001 |
| Fungus at filter area (CFU/m^3^) | 0 | (0-0) | 0 | (0-0) | 0 | (0-0) | 0 | (0-0) | 0.567 |

| **Table 3.** General characteristics of participants (n=20) | | | |  |
| --- | --- | --- | --- | --- |
| **Characteristics** |  |  |  | |
| Gender, n (%) |  |  |  | |
| Male | 16 | (80.0) |  | |
| Female | 4 | (20.0) |  | |
| Age (year), Mean±SD | 26.85 ± 7.31 | |  | |
| (Min - Max) | (19 - 44) | |  | |
| BW (kg), Mean±SD | 67.63 ± 19.99 | |  | |
| (Min - Max) | (42 - 139) | |  | |
| BMI (kg/m^2^), Mean±SD | 23.36 ± 5.13 | |  | |
| (Min - Max) | (16.41 - 40.61) | |  | |
| Mask size, n (%) |  |  |  | |
| S | 1 | (5.0) |  | |
| M | 15 | (75.0) |  | |
| L | 4 | (20.0) |  | |
| Data are presented as number (%) or mean ± standard deviation. | | | |  |

| **Table4** Face Length and Face Width |  |
| --- | --- |
| **Variable** |  |
| Face width (mm), mean ± SD | 138.55 ± 15.35 |
| (min - max) | (116 - 170) |
| Face length (mm), mean ± SD | 120.85 ± 14.34 |
| (min - max) | (96 - 150) |
| Lip length (mm), mean ± SD | 63.30 ± 13.71 |
| (min - max) | (45 - 80) |

| **Table 5** Percentage of Population and Number of Subjects for the Panel Based on Face Length and Face Width | | | | | | |
| --- | --- | --- | --- | --- | --- | --- |
| **Cell** | **Male** | | **Female** | | **Total** | |
|  | **n** | **(%)** | **n** | **(%)** | **n** | **(%)** |
| 1 | 1 | (6.3) | 2 | (50.0) | 3 | (15.0) |
| 2 | 0 | (0.0) | 0 | (0.0) | 0 | (0.0) |
| 3 | 1 | (6.3) | 0 | (0.0) | 1 | (5.0) |
| 4 | 0 | (0.0) | 1 | (25.0) | 1 | (5.0) |
| 5 | 0 | (0.0) | 0 | (0.0) | 0 | (0.0) |
| 6 | 1 | (6.3) | 0 | (0.0) | 1 | (5.0) |
| 7 | 3 | (18.7) | 0 | (0.0) | 3 | (15.0) |
| 8 | 0 | (0.0) | 0 | (0.0) | 0 | (0.0) |
| 9 | 2 | (12.5) | 0 | (0.0) | 2 | (10.0) |
| 10 | 3 | (18.7) | 0 | (0.0) | 3 | (15.0) |
| Total | 11 | (68.8) | 3 | (75.0) | 14 | (70.0) |

| **No.** | **Consent Date** | **Gender** | **Age** | **Height(cm)** | **Height(m)** | **BW(kg)** | **BMI** | **Face dimension (cm)** | | | **mask size** | No.of spray | **การทดสอบ/(ครั้ง)** | | | | | | Remark |
| --- | --- | --- | --- | --- | --- | --- | --- | --- | --- | --- | --- | --- | --- | --- | --- | --- | --- | --- | --- |
|  |  |  |  |  |  |  |  | **Face width** | **Lip length** | **Face length** |  |  | 1st | | 2nd | | 3rd | |  |
|  |  |  |  |  |  |  |  |  |  |  |  |  | pass/fail | Symptom | passฝfail | Correction | pass/fail | symptom |  |
| 1 | 17/4/2563 | Male | 21 | 173 | 1.73 | 52 | 17.37 | 15 | 7 | 13.5 | M | 4 | √ | – | – | – | – | – | – |
| 2 | 17/4/2563 | Male | 19 | 166 | 1.66 | 66 | 23.95 | 16.5 | 6 | 15 | M | 7 | √ | – | – | – | – | – | – |
| 3 | 20/4/2563 | Female | 21 | 160 | 1.6 | 42 | 16.41 | 12.5 | 4.5 | 10.2 | S | 5 | √ | – | – | – | – | – | – |
| 4 | 20/4/2563 | Male | 22 | 180 | 1.8 | 67 | 20.68 | 15 | 6 | 13 | L | 9 | √ | – | – | – | – | – | – |
| 5 | 20/4/2563 | Female | 22 | 163 | 1.63 | 61 | 22.96 | 12 | 5 | 11 | M | 5 | √ | – | – | – | – | – | – |
| 6 | 20/4/2563 | Female | 21 | 158 | 1.58 | 48 | 19.23 | 13.5 | 5 | 11 | M | 5 | √ | – | – | – | – | – | – |
| 7 | 20/4/2563 | Female | 43 | 158 | 1.58 | 51 | 20.43 | 12.5 | 4.5 | 10.5 | M | 5 | √ | – | – | – | – | – | – |
| 8 | 20/4/2563 | Male | 32 | 165 | 1.65 | 80 | 29.38 | 14 | 8 | 12 | M | 15 | √ | – | – | – | – | – | – |
| 9 | 20/4/2563 | Male | 20 | 185 | 1.85 | 139 | 40.61 | 17 | 8 | 14 | L | 12 | √ | – | – | – | – | – | – |
| 10 | 21/4/2563 | Male | 29 | 172 | 1.72 | 67 | 22.65 | 11.6 | 5.2 | 10.8 | M | 6 | √ | – | – | – | – | – | – |
| 11 | 21/4/2563 | Male | 30 | 185 | 1.85 | 85 | 24.84 | 13.5 | 7.4 | 13.2 | L | 9 | √ | – | – | – | – | – | – |
| 12 | 21/4/2563 | Male | 28 | 170 | 1.7 | 65 | 22.49 | 12.8 | 5 | 9.6 | M | 27 | √ | – | – | – | – | – | – |
| 13 | 21/4/2563 | Male | 20 | 165 | 1.65 | 65 | 23.88 | 13 | 8 | 12 | M | 5 | √ | – | – | – | – | – | – |
| 14 | 21/4/2563 | Male | 19 | 163 | 1.63 | 59 | 22.21 | 14 | 8 | 12 | M | 20 | √ | – | – | – | – | – | – |
| 15 | 21/4/2563 | Male | 25 | 173 | 1.73 | 78 | 26.06 | 16 | 7 | 13 | M | 5 | √ | – | – | – | – | – | – |
| 16 | 21/4/2563 | Male | 31 | 168 | 1.68 | 78 | 27.64 | 12.2 | 5.2 | 10.7 | L | 10 | √ | – | – | – | – | – | – |
| 17 | 21/4/2563 | Male | 44 | 168 | 1.68 | 66 | 23.38 | 12.5 | 4.5 | 11.2 | M | 10 | √ | – | – | – | – | – | – |
| 18 | 21/4/2563 | Male | 29 | 170 | 1.7 | 60.5 | 20.93 | 14.5 | 6.8 | 12.5 | M | 4 | √ | – | – | – | – | – | – |
| 19 | 21/4/2563 | Male | 30 | 175 | 1.75 | 62 | 20.24 | 14 | 7.5 | 130 | M | 21 | √ | – | – | – | – | – | – |
| 20 | 21/4/2563 | Male | 31 | 167 | 1.67 | 61 | 21.87 | 15 | 8 | 13.5 | M | 6 | √ | – | – | – | – | – | – |

| **id** | **date** | **gender** | **age** | **height_cm** | **height_m** | **bw** | **bmi** | **width** | **length_lip** | **length** | **size** | **spray** | **test1** |
| --- | --- | --- | --- | --- | --- | --- | --- | --- | --- | --- | --- | --- | --- |
| 1 | 17/4/2563 | Male | 21 | 173 | 1.73 | 52 | 17.37 | 15 | 7 | 13.5 | M | 4 | 1 |
| 2 | 17/4/2563 | Male | 19 | 166 | 1.66 | 66 | 23.95 | 16.5 | 6 | 15 | M | 7 | 1 |
| 3 | 20/4/2563 | Female | 21 | 160 | 1.6 | 42 | 16.41 | 12.5 | 4.5 | 10.2 | S | 5 | 1 |
| 4 | 20/4/2563 | Male | 22 | 180 | 1.8 | 67 | 20.68 | 15 | 6 | 13 | L | 9 | 1 |
| 5 | 20/4/2563 | Female | 22 | 163 | 1.63 | 61 | 22.96 | 12 | 5 | 11 | M | 5 | 1 |
| 6 | 20/4/2563 | Female | 21 | 158 | 1.58 | 48 | 19.23 | 13.5 | 5 | 11 | M | 5 | 1 |
| 7 | 20/4/2563 | Female | 43 | 158 | 1.58 | 51 | 20.43 | 12.5 | 4.5 | 10.5 | M | 5 | 1 |
| 8 | 20/4/2563 | Male | 32 | 165 | 1.65 | 80 | 29.38 | 14 | 8 | 12 | M | 15 | 1 |
| 9 | 20/4/2563 | Male | 20 | 185 | 1.85 | 139 | 40.61 | 17 | 8 | 14 | L | 12 | 1 |
| 10 | 21/4/2563 | Male | 29 | 172 | 1.72 | 67 | 22.65 | 11.6 | 5.2 | 10.8 | M | 6 | 1 |
| 11 | 21/4/2563 | Male | 30 | 185 | 1.85 | 85 | 24.84 | 13.5 | 7.4 | 13.2 | L | 9 | 1 |
| 12 | 21/4/2563 | Male | 28 | 170 | 1.7 | 65 | 22.49 | 12.8 | 5 | 9.6 | M | 27 | 1 |
| 13 | 21/4/2563 | Male | 20 | 165 | 1.65 | 65 | 23.88 | 13 | 8 | 12 | M | 5 | 1 |
| 14 | 21/4/2563 | Male | 19 | 163 | 1.63 | 59 | 22.21 | 14 | 8 | 12 | M | 20 | 1 |
| 15 | 21/4/2563 | Male | 25 | 173 | 1.73 | 78 | 26.06 | 16 | 7 | 13 | M | 5 | 1 |
| 16 | 21/4/2563 | Male | 31 | 168 | 1.68 | 78 | 27.64 | 12.2 | 5.2 | 10.7 | L | 10 | 1 |
| 17 | 21/4/2563 | Male | 44 | 168 | 1.68 | 66 | 23.38 | 12.5 | 4.5 | 11.2 | M | 10 | 1 |
| 18 | 21/4/2563 | Male | 29 | 170 | 1.7 | 60.5 | 20.93 | 14.5 | 6.8 | 12.5 | M | 4 | 1 |
| 19 | 21/4/2563 | Male | 30 | 175 | 1.75 | 62 | 20.24 | 14 | 7.5 | 130 | M | 21 | 1 |
| 20 | 21/4/2563 | Male | 31 | 167 | 1.67 | 61 | 21.87 | 15 | 8 | 13.5 | M | 6 | 1 |

| id | Identification number | No. |  |  | la var id "Identification number" | | | |  | |
| --- | --- | --- | --- | --- | --- | --- | --- | --- | --- | --- |
| name | Name Surname | Name Surname |  |  | la var name "Name Surname" | | | |  | |
| date | Consent Date | Consent Date |  |  | la var date "Consent Date " | | | |  | |
| gender | Gender | Gender |  |  | la var gender "Gender" | | | |  | |
| age | Age | Age |  |  | la var age "Age" | |  | |  | |
| height_cm | Height (cm) | Height(cm) |  |  | la var height_cm "Height (cm)" | | | |  | |
| height_m | Height (m) | Height(m) |  |  | la var height_m "Height (m)" | | | |  | |
| bw | BW (kg) | BW(kg) |  |  | la var bw "BW (kg)" | |  | |  | |
| bmi | BMI | BMI |  |  | la var bmi "BMI" | |  | |  | |
| width | Face width (cm) | face dimension (cm) | Face width | | la var width "Face width (cm)" | | | |  | |
| length_lip | Lip length (cm) |  | Lip length |  | la var length_lip "Lip length (cm)" | | | | | |
| length | Face length (cm) |  | Face length | | la var length "Face length (cm)" | | | |  | |
| size | Mask size | mask size | | | | la var size "Mask size" | | | |  |
| spray | Spray | no.of test | | | | la var spray "Spray" | |  | |  |
| test1 | Test 1st | no.of test | 1st | pass/fail | la var test1 "Test 1st" | | | |  | |
| character1 | Characteristic 1st |  |  | symptoms | la var character1 "Characteristic 1st" | | | | | |
| test2 | Test 2nd |  | 2nd | pass/fail | la var test2 "Test 2nd" | | | |  | |
| character2 | Characteristic 2nd |  |  | correcting method | la var character2 "Characteristic 2nd" | | | | | |
| test3 | Test 3rd |  | 3rd | pass/fail | la var test3 "Test 3rd" | | | |  | |
| character3 | Characteristic 3rd |  |  | symptoms | la var character3 "Characteristic 3rd" | | | | | |
| note | Note | Remark | | | | la var note "Note" | |  | |  |
