# Supplementary material for: Impact of ultraviolet germicidal irradiation on new silicone half-piece elastometric respirator (VJR-NMU) performance, structural integrity and sterility during the COVID-19 pandemic
Source: PLoS One. 2021 Oct 14;16(10):e0258245. doi: 10.1371/journal.pone.0258245 (PMC8516203; doi:10.1371/journal.pone.0258245)
Supplement: Attachment — Submitted filename: Response to the comment reuse N99.docx [file pone.0258245.s008.docx]

**RESPONSE TO REVIEWER COMMENTS AND SUGGESTIONS**

Thank you for the opportunity to respond to the editors’ and reviewers’ comments on our revised manuscript. Our responses are provided after each comment. We hope that these extensive changes, based on their critiques, make this manuscript suitable for publication in PLoS one**.**

**COMMENTS FOR THE AUTHOR:**

1. Please ensure that your manuscript meets PLOS ONE's style requirements, including those for file naming.

**Authors’ response:** Thank you for your comments. We have revised the manuscript style according to the journal’s requirement.

2. Please review your reference list to ensure that it is complete and correct.

**Authors’ response:** Thank you for your comments**.**We reviewed all the references and found no retracted references.

3. Your reasons for your delay in registering this study (after enrolment of participants started)

**Authors’ response:** Thank you for your comments. The registration was done after enrolment of participants due to the difficulty of the viral culture in our lab and the possibility of changing the protocol.After adjustment and improvement in nutrient media and uneventful protocol,the registration was performed. We already described the reason for the delay registration in the manuscript.

4. We note that you have stated that you will provide repository information for your data at acceptance. Should your manuscript be accepted for publication, we will hold it until you provide the relevant accession numbers or DOIs necessary to access your data.

**Authors’ response** : Thank you for your comments.The repository data was added in the Appendix section in the supplemental file.We will make change to our Data Availability statement in the cover letter.

5.PLOS ONE now requires that authors provide the original uncropped and unadjusted images underlying all blot or gel results reported in a submission’s figures or Supporting Information files.

**Authors’ response** : Thank you for your comments.Almost all figures in this manuscript are photos taken from our lab.The exception are Fig 11 in the manuscript and S3 Fig in the supplemental file that will be sent together in original files.All of these changes will be mentioned in the cover letter.

6. Please include captions for your Supporting Information files at the end of your manuscript, and update any in-text citations to match accordingly.

**Authors’ response** : Thank you for your comments.All the captions were added at the end of the manuscript and cited in the text to match accordingly.

**Review Comments to the Author**

- Is it better to use a new HEPA Filter if it is available?

**Authors’ response**

Thank you for your comments If there is enough and available new HEPA Filter, it is definitely better to use the new one.However,due to the high cost of the HEPA filter and short supply,we try to find the easiest and safe way to decontaminate them.
- Alcohol 70 % may affect on the effectiveness of HEPA filter

**Authors’ response**

Thank you for your comments.
HEPA filters are made of thin fibers of glass, and contain some level of activated carbon-based material.Therefore it should not be affected by alcohol 70%/
- It is difficult to remove accumulated fine particles in HEPA filter

**Authors’ response**

Thank you for your comments.
If the particulate is dense ,removal will be difficult especially with the HEPA filter from SafeStar (Draeger,Germany).However,our another experiment with new version of our HEPA filter showed that the HEPA filter can be used up to one month and the accumulated fine particles are easily removed

| Day | Air flow (LPM) | Filtration efficiency (%) |
| --- | --- | --- |
| 1 | 320.56 | 99.71 |
| 1 | 320.56 | 99.81 |
| 2 | 335.83 | 99.75 |
| 3 | 335.83 | 99.55 |
| 4 | 335.83 | 99.38 |
| 5 | 351.09 | 99.87 |
| 6 | 335.83 | 99.86 |
| 7 | 335.83 | 99.94 |
| 8 | 305.30 | 99.97 |
| 9 | 335.83 | 99.89 |
| 10 | 274.77 | 99.67 |
| 11 | 259.50 | 99.65 |
| 12 | 259.50 | 99.52 |
| 13 | 259.50 | 99.39 |
| 14 | 274.77 | 99.84 |
| 15 | 236.61 | 99.79 |
| 16 | 224.393844 | 99.96 |
| 17 | 222.8673553 | 99.91 |
| 18 | 221.3408665 | 99.87 |
| 19 | 219.8143778 | 99.86 |
| 20 | 219.8143778 | 99.90 |
| 21 | 219.8143778 | 99.83 |
| 22 | 219.8143778 | 99.89 |
| 23 | 219.8143778 | 99.85 |
| 24 | 219.8143778 | 99.80 |
| 25 | 219.8143778 | 99.87 |
| 26 | 219.8143778 | 99.92 |
| 27 | 210.6554454 | 99.85 |
| 28 | 210.6554454 | 99.90 |
| 29 | 210.6554454 | 99.87 |
| 30 | 210.6554454 | 99.76 |
|  |  |  |

Filtraion efficiency of N99

|  | อัตราการไหลของอากาศ (LPM) | ความเร็วลม (m/s) | ประสิทธิภาพการกรอง (%) |
| --- | --- | --- | --- |
| 1 44003 | 320.563 | 21 | 99.7067 |
| 1 44004 | 320.563 | 21 | 99.8067 |
| 2 44004 | 335.828 | 22 | 99.7533 |
| 3 44005 | 335.828 | 22 | 99.5533 |
| 4 44006 | 335.828 | 22 | 99.3833 |
| 5 44007 | 351.092 | 23 | 99.87 |
| 6 44008 | 335.828 | 22 | 99.8567 |
| 7 44009 | 335.828 | 22 | 99.9433 |
| 8 44010 | 305.298 | 20 | 99.9667 |
| 9 44011 | 335.828 | 22 | 99.8933 |
| 10 44012 | 274.768 | 18 | 99.67 |
| 11 44013 | 259.503 | 17 | 99.6533 |
| 12 44014 | 259.503 | 17 | 99.5233 |
| 13 44015 | 259.503 | 17 | 99.3933 |
| 14 44016 | 274.768 | 18 | 99.84 |
| 15 44017 | 236.606 | 15.5 | 99.79 |
| 16 44018 | 224.394 | 14.7 | 99.9567 |
| 17 44019 | 222.867 | 14.6 | 99.9133 |
| 18 44020 | 221.341 | 14.5 | 99.87 |
| 19 44021 | 219.814 | 14.4 | 99.8567 |
| 20 44022 | 219.814 | 14.4 | 99.9033 |
| 21 44023 | 219.814 | 14.4 | 99.8333 |
| 22 44024 | 219.814 | 14.4 | 99.8933 |
| 23 44025 | 219.814 | 14.4 | 99.8467 |
| 24 44026 | 219.814 | 14.4 | 99.8 |
| 25 44027 | 219.814 | 14.4 | 99.8733 |
| 26 44028 | 219.814 | 14.4 | 99.9167 |
| 27 44029 | 210.655 | 13.8 | 99.8533 |
| 28 44030 | 210.655 | 13.8 | 99.8967 |
| 29 44031 | 210.655 | 13.8 | 99.87 |
| 30 44032 | 210.655 | 13.8 | 99.7567 |

I hope this revision will answer and fulfil the reviewer and the Editor’s suggestion.

Sincerely,

Thananda Trakarnvanich M.D.
